# A Case Report on the Diagnosis and Management of a Rare Ameloblastic Fibro-Odontoma in the Anterior Maxilla of a Pediatric Patient

**DOI:** 10.7759/cureus.84343

**Published:** 2025-05-18

**Authors:** Hiralal Ash, Rajeev Kumar Singh, Eram Anwar, Abhishek Banerjee, Ananjan Chatterjee, Karthikeyan Ramalingam

**Affiliations:** 1 Oral and Maxillofacial Surgery, Buddha Institute of Dental Sciences and Hospital, Patna, IND; 2 Oral and Maxillofacial Pathology, Awadh Dental College and Hospital, Jamshedpur, IND; 3 Oral and Maxillofacial Pathology, Buddha Institute of Dental Sciences and Hospital, Patna, IND; 4 Oral Pathology and Microbiology, Malla Reddy Institute of Dental Sciences, Malla Reddy Vishwavidyapeeth, Hyderabad, IND

**Keywords:** ameloblastic fibro-odontoma, ameloblastoma, benign odontogenic neoplasms, child, dental tumors, jaw neoplasms, maxillary neoplasms, neoplasm, odontogenic tumors, pediatric dentistry

## Abstract

Ameloblastic fibro-odontoma (AFO) is an uncommon, non-cancerous odontogenic tumor that predominantly affects children and young adolescents. This case report details a nine-year-old male patient who presented with a firm swelling accompanied by intermittent serous discharge in the upper right jaw following a fall. Clinical assessment revealed incomplete mouth closure, facial asymmetry, and dentoalveolar extrusion. Radiographic analysis via cone beam computed tomography revealed a heterogeneous radiolucent lesion with focal radiopacities, thinning of the cortical plates, and a minor breach in the nasal floor, indicative of an osteolytic lesion.

Differential diagnoses considered included adenomatoid odontogenic tumor and desmoplastic ameloblastoma. Routine blood tests and fine-needle aspiration cytology (FNAC) yielded negative results. The lesion was surgically enucleated under general anesthesia, with preservation of the affected teeth. Histopathological analysis confirmed the diagnosis of AFO, characterized by hyperchromatic columnar ameloblast-like cells, stellate reticulum-like cells, and basophilic dentinoid-like formation within an immature connective tissue stroma. Postoperative recovery was smooth, resulting in notable improvement in facial aesthetics and oral function.

AFO is typically located in the posterior mandible, making its occurrence in the anterior maxilla unusual. Conservative surgical management is the recommended strategy and is associated with low recurrence rates. Although malignant transformation has been documented, extensive treatment is generally reserved for cases exhibiting dysplastic changes or aggressive recurrence. This case underscores the importance of early diagnosis, imaging, and histological verification for effective treatment planning. Regular follow-up is crucial to monitor for potential recurrence and to ensure favorable long-term outcomes in patients with AFO.

## Introduction

Ameloblastic fibro-odontoma (AFO) is a rare benign odontogenic tumor. It shares features with ameloblastic fibroma while exhibiting inductive changes that lead to enamel and dentin formation [1]. AFO accounts for approximately 1-3% of odontogenic tumors, with a reported prevalence of up to 10% in patients younger than 10 years [2].

AFO primarily affects individuals under 20 years of age, with an average onset around 11.5 years. The condition exhibits a male predominance and is most commonly encountered in the posterior mandible, with less frequent involvement of the maxilla [3, 4]. AFO usually presents as a painless, slow-growing lesion that may result in swelling and delayed tooth eruption, often associated with an unerupted tooth. Radiographically, it appears as a well-defined radiolucent lesion containing radiopaque foci, typically measuring around 2 cm, although larger lesions may cause facial deformity [5, 6]. The presence of hard tissue formation distinguishes AFO from conventional ameloblastic fibroma. Various researchers have also suggested that AFO may represent a transitional stage between ameloblastic fibroma and odontoma [5].

Histologically, AFO is characterized by odontogenic epithelium within a highly cellular ectomesenchymal stroma and often contains structures such as osteodentin, dentin-like material, and enamel matrix. Treatment generally involves a conservative surgical approach, i.e., enucleation, sometimes accompanied by removal of the associated tooth. The prognosis is highly favorable, with minimal risk of recurrence [5, 6].

## Case presentation

**Figure 1:**
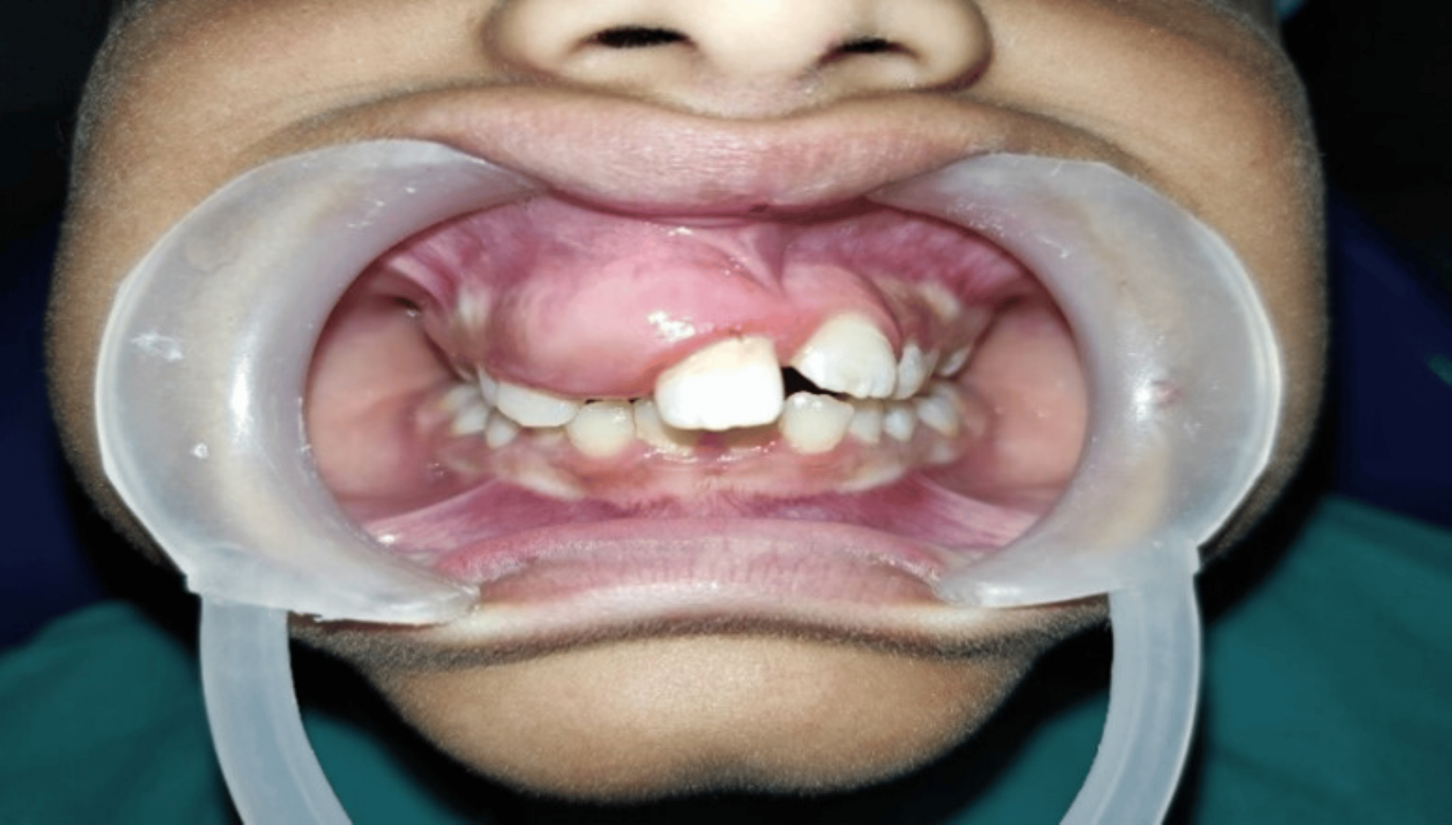
Clinical image showing maxillary swelling with associated tooth displacement.

A 9-year-old male patient reported to our OPD with swelling and watery discharge from the upper right jaw for the past year following a fall while skating, which had loosened his anterior milk teeth. As the permanent teeth erupted, a hard swelling developed, gradually increasing in size and accompanied by intermittent serous discharge. Facial asymmetry was noted, with deviation toward the left. A non-tender, hard swelling measuring approximately 1 cm × 1 cm was observed, extending from the right philtral dimple to the right corner of the lip. The right ala of the nose appeared stretched, and the patient exhibited incomplete mouth closure, leading to angular cheilitis at the left lip commissure. The patient was in the mixed dentition stage, with an unerupted permanent canine. A non-fluctuant, hard, tender swelling was causing dentoalveolar extrusion of the maxillary anterior teeth and stretching of the labial frenum. A midline shift with a diastema was noted between the maxillary incisors (Figure 1).

**Figure 2:**
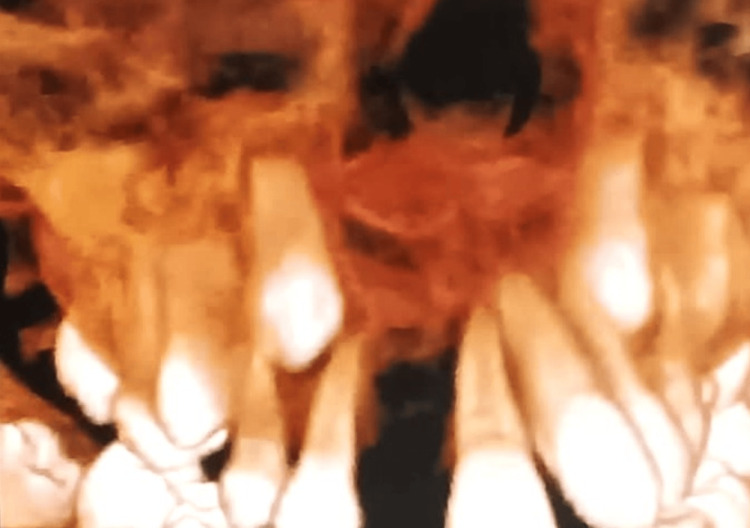
CBCT image showing a non-homogeneous lesion involving the anterior maxilla. CBCT: Cone-beam computed tomography.

**Figure 3:**
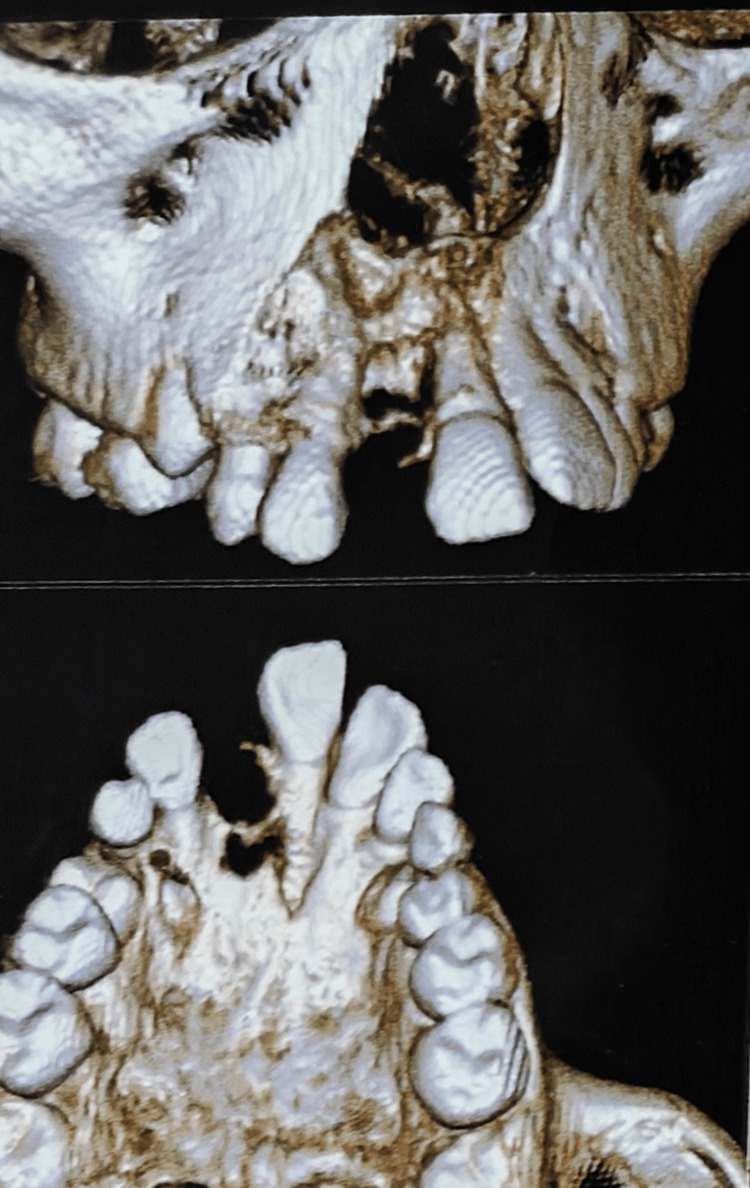
CBCT images showing bone destruction in the anterior maxilla. CBCT: Cone-beam computed tomography.

Cone beam CT revealed a non-homogeneous radiolucency measuring 21.77 mm × 17.11 mm × 12.77 mm, containing radiopaque entities. There was thinning of the cortical plates and a slight breach in the nasal floor, suggesting a provisional diagnosis of an osteolytic lesion (Figures 2-3). 

**Figure 4:**
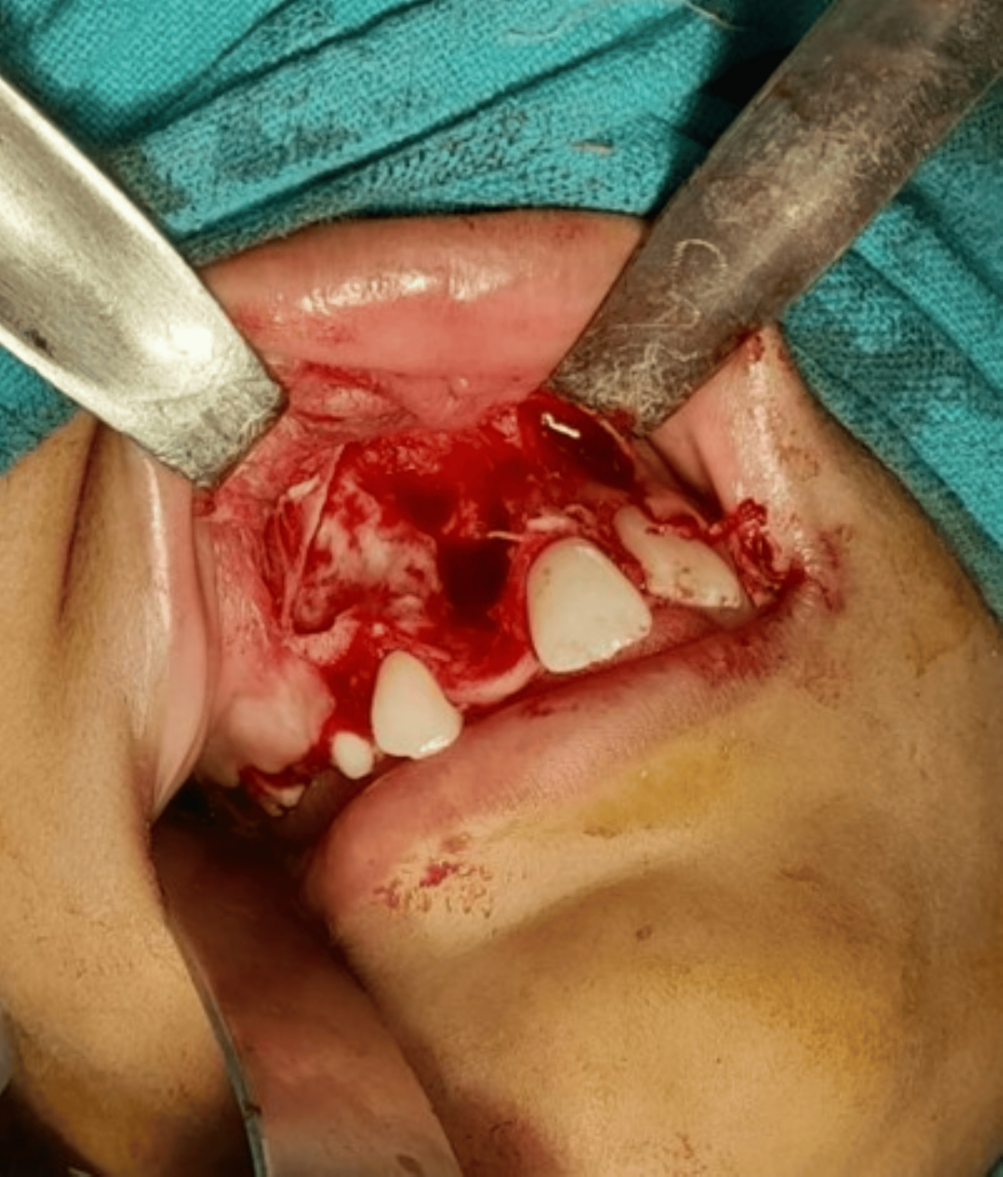
Clinical photograph after surgical curettage of the anterior maxilla.

The differential diagnoses included adenomatoid odontogenic tumor, ameloblastoma, central giant cell granuloma, and ossifying fibroma. Routine blood investigations and fine needle aspiration cytology (FNAC) were performed, both yielding negative results. Enucleation of the lesion was planned under general anesthesia. A mid-crestal incision was made, and a trapezoidal flap was reflected to access the lesion. Thin, fragile bone was removed, and the mass (2 cm × 1 cm) was extracted. Curettage and osteoplasty were performed, and the cavity was irrigated before suturing. The extruded tooth and unerupted canine were preserved (Figure 4).

**Figure 5:**
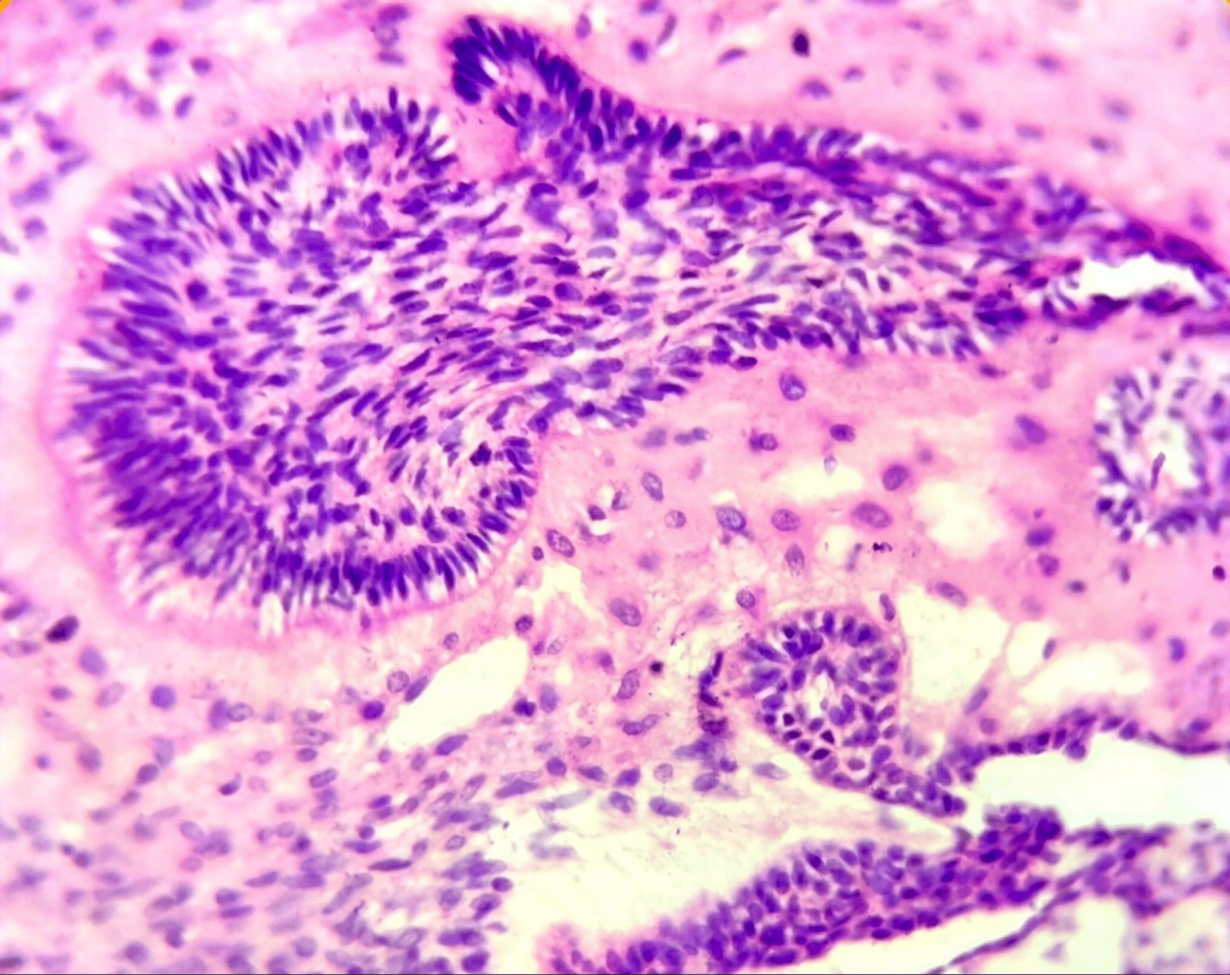
Photomicrograph showing ameloblast-like cells with reversal of nuclear polarity, along with centrally placed stellate reticulum-like cells arranged in nests (H&E, 10×).

Histopathological examination revealed lesional tissue consisting of hypercellular areas and an immature connective tissue stroma. The hypercellular areas comprised cords and strands of hyperchromatic, columnar ameloblast-like cells with reversal of nuclear polarity, along with centrally placed stellate reticulum-like cells arranged in nests (Figure 5). 

**Figure 6:**
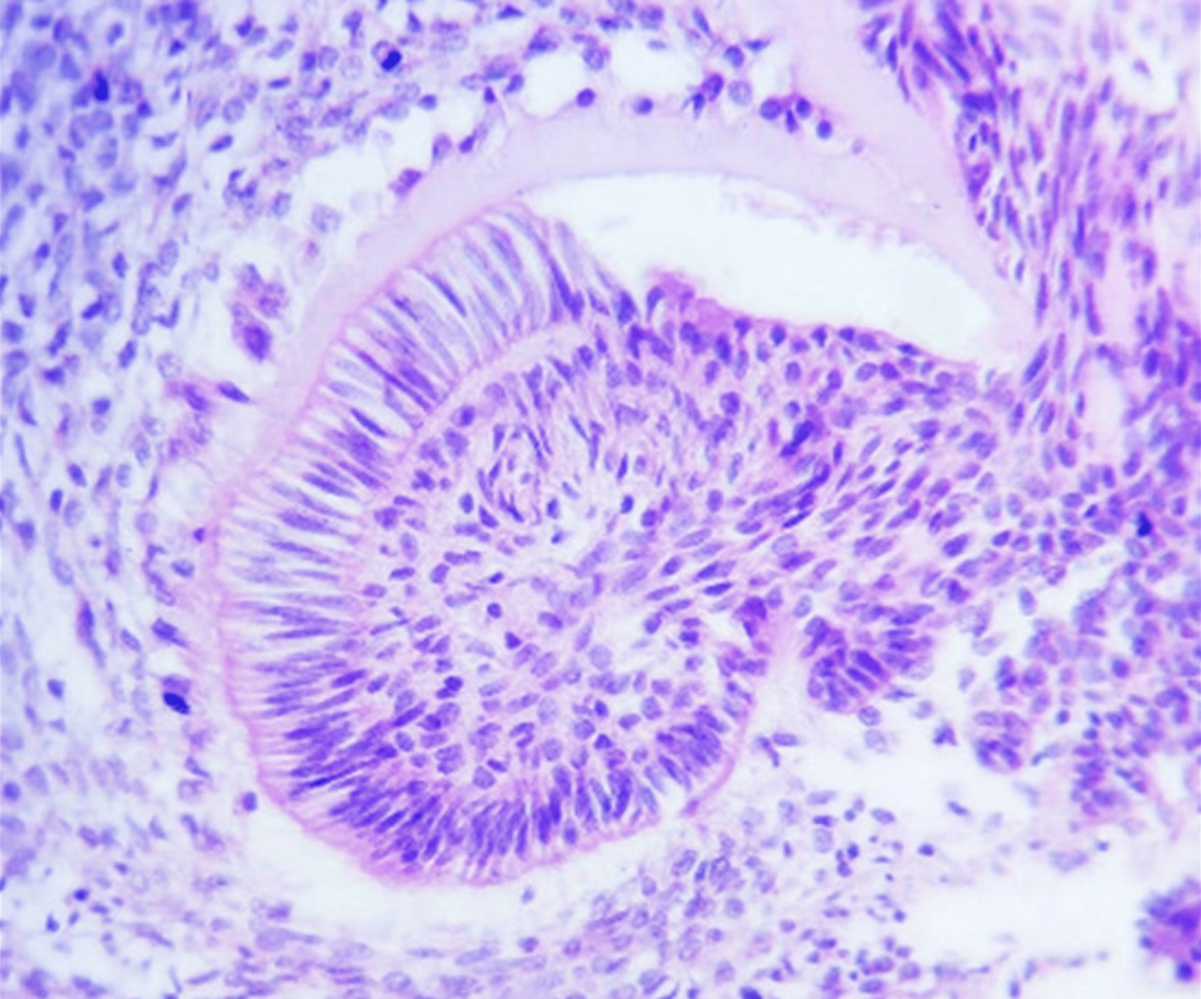
Photomicrograph showing focal areas of basophilic dentinoid-like formation (H&E, 20×).

Focal areas of basophilic dentinoid-like formation were observed. The stromal component was immature, resembling dental papilla and composed of stellate-shaped fibroblasts (Figure 6), confirming a diagnosis of AFO.

**Figure 7:**
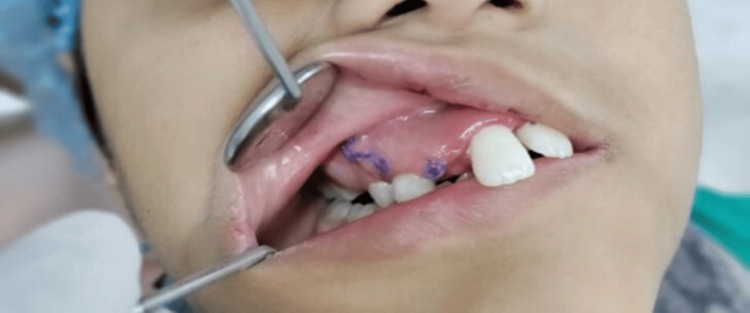
Clinical photograph showing postoperative follow-up.

The postoperative course after curettage was uneventful; there was significant improvement in aesthetics, with edema subsiding and the alveolus beginning to intrude. The anterior teeth (central and lateral incisors) remained tender (Figure 7). The patient was scheduled for follow-up appointments to monitor healing and ensure recovery proceeded without complications. He was satisfied with the postoperative outcome in facial appearance and oral function.

## Discussion

**Figure 8:**
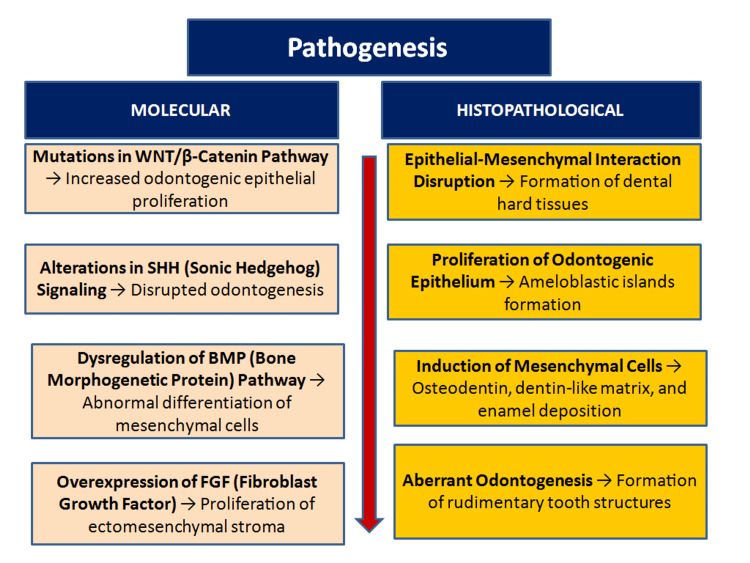
Infographic illustrating the pathogenesis of ameloblastic fibro-odontoma. Image content was created by the authors.

AFO is a rare, benign odontogenic tumor commonly affecting children. It was first differentiated from ameloblastic odontoma by Hooker. Debate persists regarding whether ameloblastic fibroma, AFO, and odontoma represent stages in a developmental continuum or are distinct entities [7]. Regezi suggested that AFO is a derivative of ameloblastic fibroma, while Slootweg described AFO as an immature form of complex odontoma. The pathogenesis of AFO remains unclear, but it is believed that mutations in the WNT (Wingless-related integration site signaling pathway)/β-catenin pathway lead to increased odontogenic epithelial proliferation. Alterations in Sonic Hedgehog (SHH) signaling result in disrupted odontogenesis, while dysregulation of Bone Morphogenetic Protein (BMP) is associated with abnormal differentiation of mesenchymal cells. Additionally, overexpression of Fibroblast Growth Factor (FGF) contributes to the proliferation of ectomesenchymal stroma (Figure 8) [7, 8]. Neoplastic behavior and malignant transformation of AFO have also been reported by Howell RM and Burkes EJ Jr [8].

The most common clinical presentations of AFO include asymptomatic swelling and failure of tooth eruption [3, 7, 8]. Studies indicate that AFO most frequently occurs in the posterior mandible (60% of cases), with the anterior maxilla being the least commonly affected site (7% of cases) [9]. Radiographically, the lesion appears as a well-circumscribed, expansile radiolucency that often contains foci or numerous small radiopaque areas [10]. Larger areas of calcification can make it difficult to distinguish AFO from complex odontoma radiographically. The differential diagnoses include lesions with mixed radiographic features, such as immature calcifying epithelial odontogenic tumor, ameloblastoma, adenomatoid odontogenic tumor, and fibro-osseous lesions [7]. Histopathologically, the absence of duct-like structures and rosette patterns helped us to exclude adenomatoid odontogenic tumor. Desmoplastic ameloblastoma was also ruled out, as the stroma of the tumor was very immature and resembled ectomesenchyme. The absence of giant cells and hemorrhagic areas led us to exclude central giant cell lesions as well [7].

The literature suggests that AFO is typically treated using a conservative surgical approach [4]. Recurrences have been reported, often due to inadequate surgical removal during initial treatment [10]. Controversy exists regarding whether to extract or retain the associated tooth bud in AFO cases. Some authors advocate for removal to prevent recurrence [11, 12]. AFO can present with varied clinical and radiological features in both pediatric and adult patients [3]. While localized cases can be managed with enucleation and osteoplasty, more extensive surgery is required for tumors with expansile growth in adults [13].

A few cases of maxillary AFO with facial swelling and visual disturbances have been described. Lesions involving the maxilla and zygoma tend to be more extensive and may necessitate complex surgeries, including wide excision and reconstruction with an obturator [3, 4]. Some authors recommend that if the tumor is associated with foul-smelling discharge, it should first be treated with antibiotics before proceeding to aggressive interventions, such as maxillectomy or partial maxillectomy with osteocutaneous free flap reconstruction under general anesthesia [14, 15].

AFO is a rare mixed odontogenic tumor, with its incidence in the literature reported to be between 0.3% and 1.7% of all odontogenic tumors. This tumor is also more commonly found in the mandibular posterior region, which makes our case notable as it highlights occurrence in the maxillary anterior region. Along with early diagnosis, imaging, and appropriate surgical intervention, good prognostic outcomes can be achieved. This case report emphasizes the importance of these factors [15, 16].

Since the tumor in our patient was relatively small and radiographic findings did not indicate extensive destruction, a conservative approach was deemed appropriate. Hence, we present this case along with a review of reported cases, focusing on tumor location and treatment rendered (Table 1).

**Table 1:** Reported cases of ameloblastic fibro-odontoma (2015-2025): a decade review.

Year	Author(s)	Age	Gender	Location	Clinical Presentation	Treatment
2015	Gantala R et al. [16]	11	Female	Left posterior mandible	Swelling	Enucleation, osteoplasty
2019	Prakash Rao et al. [17]	14	Female	Right posterior mandible	Swelling, unerupted tooth	Surgical removal
2020	Cossiez M et al. [18]	9	Male	Maxilla	Delayed eruption of tooth 26	Enucleation
2021	Divya B et al. [19]	13	Female	Right posterior mandible	Swelling, delayed tooth eruption	Conservative enucleation
2022	Julya VADS et al. [20]	8	Male	Mandible	Asymptomatic lesion	Enucleation
2023	Jihed S et al. [21]	37	Female	Anterior mandible	Swelling	Surgical excision
2024	Kumar M et al. [22]	15	Male	Left mandible	Swelling	Surgical excision
2025	Present case	9	Male	Upper right maxilla	Swelling, watery discharge	Enucleation, osteoplasty

AFO recurrence has also been associated with malignant transformation. However, many authors suggest that this does not necessitate radical treatment for all benign lesions. Instead, if recurrence occurs with histological evidence of dysplastic changes, only then should more extensive procedures be considered [8, 14, 16].

## Conclusions

AFO typically presents with minimal clinical symptoms and carries a favorable prognosis. Early diagnosis, confirmed through histological examination, is essential for effective management. Conservative treatment, such as enucleation of the lesion with preservation of the associated teeth, is usually sufficient and is associated with a low recurrence rate. Supportive measures, including the use of titanium plates and bone grafts, can help maintain structural integrity during recovery. Although AFO generally has a good long-term outlook, regular follow-up is crucial to monitor for potential recurrence or complications. Timely intervention and appropriate care contribute significantly to successful clinical outcomes.
